# Interferon Regulatory Factor 6 Has a Protective Role in the Host Response to Endotoxic Shock

**DOI:** 10.1371/journal.pone.0152385

**Published:** 2016-04-01

**Authors:** Sophie Joly, Lindsey Rhea, Paige Volk, Jessica G. Moreland, Martine Dunnwald

**Affiliations:** 1 Department of Internal Medicine, The University of Iowa, Iowa City, IA, United States of America; 2 Department of Pediatrics, The University of Iowa, Iowa City, IA, United States of America; Karolinska Institutet, SWEDEN

## Abstract

Interferon Regulatory Factor (IRF) 6, a member of the IRF family, is essential for epidermal and orofacial embryonic development. Irf6 is strongly expressed in keratinocytes, in which it regulates epidermal proliferation, differentiation, and migration. A recent role for Irf6 in Toll-like receptor 2-dependent chemokine gene expression was also reported in an epithelial cell line. However, a function for Irf6 in innate immune cells was not previously reported. In the present study, we investigated the expression and function of Irf6 in bone marrow-derived neutrophils and macrophages. We show here, using a conditional knockout of Irf6 in lysosymeM expressing cells, that Irf6 is required for resistance to LPS-induced endotoxic shock. In addition, Irf6-deficient bone marrow-derived neutrophils exhibited increased chemotactic index and velocity compared with wild-type cells *in vitro*. TLR4-specific KC and IL6 secretions were upregulated in Irf6-deficient bone marrow-derived macrophages *in vitro*. These cells also exhibited an increased level of phosphorylated IkBa. Collectively, our findings suggest a role for Irf6 in the resistance to endotoxic shock due to NFk-B-mediated alteration of cytokine production.

## Introduction

The Interferon Regulatory Factor (IRF) family of transcription factors comprises 9 family members (IRF1 to 9) that are characterized by a conserved amino (N)-terminal DNA-binding domain forming a helix-turn-helix domain [1]. The carboxy-terminal domain exhibits increased diversity and confers specificity to each of the IRF family members. Although IRFs were initially described as mediating the interferon response after viral infections [1], this family is now recognized as playing a wider role in embryonic development, health and diseases like cancer [2, 3], metabolism and traumatic brain injury [4]. Of particular interest is the involvement of multiple family members in the regulation of immune responses and immune cell development (for review, [5]). IRF1 and IRF2 are required for the development of NK cells, whereas IRF4 and IRF8 are involved in the differentiation of B-cells as well as the development of myeloid and dendritic cells. IRF3, IRF5, and IRF7, on the other hand, are involved in the regulation of innate immune responses including mediation of the Toll-like receptor pathway [6–8].

Interferon Regulatory Factor 6 is a unique family member. First, its structure resembles the one of IRF3, yet it has a unique carboxy-terminal region [9]. Irf6 is detected in a wide variety of organs, including the placenta, the kidney, and the liver [10]. It is strongly expressed in epithelial cells, particularly the mammary [11] and oral epithelial cells [12], as well as keratinocytes [13–15]. It interacts with maspin in breast epithelial cells [16], and regulates cell cycle entry upon phosphorylation [17]. In keratinocytes, Irf6 is involved in a feedback loop with p63 to regulate epidermal differentiation *in vivo* and *in vitro* [13, 18, 19]. Furthermore, Irf6 is necessary for proper keratinocyte migration [20]. During development, Irf6 is upregulated upon the adhesion and fusion of the two palatal shelves during palatogenesis [12]. Consequently, lack of Irf6 in the mouse leads to orofacial, limb and epidermal anomalies, and is essential for life as neonatal *Irf6*-deficient mice are perinatal lethal [14, 15]. In human, mutations in *IRF6* cause two orofacial clefting syndromes, Van der Woude syndrome (VWS) and popliteal pterygium syndrome (PPS) [10]. Interestingly, patients with VWS are at higher risk for developing wound-healing complications following cleft surgical repair [21], suggesting a potential role for Irf6 in the inflammatory response after injury. Both epithelial cells and innate immune cells contribute to the inflammatory phase of tissue repair. A recent study suggests a role for Irf6 in regulating TLR2-dependent chemokine gene expression in epithelial cell lines [22]. However, nothing is known about the role of Irf6 in leukocytes and bone marrow-derived granulocytes.

In the present study, we investigated the expression and role of Irf6 in innate immune cells and immunity. We report for the first time the expression of Irf6 in neutrophils, macrophages, and dendritic cells. Furthermore, we demonstrate a role for Irf6 in the resistance to endotoxic shock due to NFk-B-mediated alteration of cytokine production.

## Material and Methods

### Animals

All animal procedures were approved by the University of Iowa Institutional Animal Care and Use Committee protocol 5011268. Male and female (8–12 weeks old) C57Bl/6, *LysM*^*Cre*^ knock-in (B6.129P2-*Lyz2*^*tm1(cre)Ifo*^/J, stock 004781;Jackson Laboratories, Bar Harbor, ME, USA), and *Irf6*^*fl*^ (generous gift from Brian Schutte, Michigan State University, USA) were used. LoxP sites were located in introns 2 and 4 of the *Irf6* gene. Recombination excised exons 3 and 4, which deleted part of the DNA binding domain. This is an *Irf6* null (nl) allele as mice that are homozygous for this deletion have the expected null phenotype (Kinoshita and Schutte, personal communication). Crosses between *Irf6*^*fl/fl*^ or *Irf6*^*fl/nl*^ and *LysM*^*Cre*^ lead to *LysM*^*Cre*^;*Irf6*^*fl/fl*^ or *LysM*^*Cre*^;*Irf6*^*fl/nl*^, named Irf6 cKO hereafter. *Irf6*^*fl/fl*^ or *Irf6*^*+/+*^ will be considered wild type.

### Isolation of bone marrow-derived cells, Toll-like Receptor stimulation, and quantitative PCR analysis

Whole bone marrow was obtained from the tibia and femur of 8–12 week old mice. Bone marrow-derived macrophages and bone marrow-derived dendritic cells were obtained after a week of *in vitro* culture as previously described [23]. Bone marrow-derived neutrophils were isolated following a ficoll gradient protocol as previously described [24]. Human neutrophils were obtained from whole blood following a similar protocol [25]. Whole blood was collected from participants who consented to the study following proper institutional review board protocol approved by the University of Iowa.

Quantitative PCR (qPCR) was performed according to a standard protocol. Primers for Irf6 (forward: *5’-CAGAGATTCCAAACGCTTCC-3’* for the detection of the null or recombined allele or *5’-TTGAGCAGTCACAGCACCAT-3’* for the detection of the wild type or non-recombined allele, reverse: *5’-GTTCTGTTTTGGGCCACACT-3’*) and B-actin (forward: *5’-GCTGATTCCCCTCCATCGT-3’*, reverse: *5’-CCTCGTCACCCACATAGGA-3’*) were used.

### Immunostaining, confocal and Western blot analysis

Cytospins of neutrophils, macrophages and dendritic cells were permeabilized with 70% acetone for 10 min at 4°C then fixed in 10% formalin for 20 min at room temperature. Cells were immunostained as previously described [13]. Primary antibodies were: rat anti-mouse CD11b (clone M1/70.15.11.5.2 developed by Dr. Springer and obtained from the Developmental Studies Hybridoma Bank under the auspices of the NICHD and maintained by the University of Iowa, Department of Biology, Iowa City, IA 52242), mouse anti-human CD11b (BD Biosciences, Franklin Lakes, NJ, USA), rabbit anti-mouse Irf6 [16], mouse anti-human CD63 (clone H5C6 developed by Dr. August and Hildreth and obtained from the Developmental Studies Hybridoma Bank under the auspices of the NICHD and maintained by the University of Iowa, Department of Biology, Iowa City, IA 52242), and Alexa Fluor 488-conjugated rat anti-mouse Ly-6G (eBioscience, San Diego, CA). Secondary antibodies were: goat anti-mouse FITC (Sigma, St-Louis, MO), and goat anti-rabbit Alexa 568 (Molecular Probes, Grand Island, NY). Microscopic observations were performed with an E800 Eclipse Nikon and photomicrographs recorded in black and white with an RT-Slider (SPOT). Human neutrophils were viewed with a Zeiss LSM 510 Meta confocal microscope using a Plan-Apochromat 63X/1.4 oil DIC objective with Zeiss Immersol 518F halogen free/fluorescence free imaging medium. Pictures were taken with an AxioCam HR digital camera, and images were acquired with LSM 510 acquisition software. The images were processed with Image J.

For IkBalpha (IkBa), phospho-IkBalpha (p-IkBa) and Irf6 detection, bone marrow-derived macrophages were incubated with 50 ng/ml lipopolysaccharides (LPS, from *E*. *Coli* 0111:B4, Sigma) and cells harvested after 30 min, 60 min, and 180 min. Proteins were extracted using Laemmli buffer, and 20 μg of protein loaded on a 10% acrylamide gel. Western blot analysis was performed as previously described [13]. Primary antibodies were: IkBa (Cell Signaling, Danvers, MA), p-IkBa (Cell Signaling), mouse anti-human beta-actin (Sigma). Secondary antibodies were: sheep anti-mouse horseradish peroxidase (HRP, GE Healthcare, Little Chalfont Buckinghamshire, UK) and donkey anti-rabbit horseradish peroxidase (Santa-Cruz Biotechnology, Dallas, TX). Western blots were visualized with a chemiluminescent HRP substrate (Pierce, Grand Island, NY) and a low-light imaging system (LAS4000, Fuji Medical Systems, Stamford, CT).

### Endotoxic shock, intraperitoneal lavage and serum collection

LPS (15 mg/kg of body weight) was injected intraperitoneally in 8–12 week old mice. For the endotoxic shock resistance assay, survival was assessed over a 7-day period. Animals were monitored three times a day for the first 3 days, than twice a day for the remaining of the experiment. Mice found in a moribund state (hunched posture, lethargy or ruffed fur) for >4 h were considered terminal and euthanized using carbon dioxide followed by cervical dislocation. For the *in vivo* neutrophil migration assay, intraperitoneal lavage was performed 8 h following LPS (15 mg/kg of body weight) injection and neutrophil migration was analyzed by flow cytometry as previously described [26]. Also, cardiac puncture was performed to collect blood serum for cytokine measurements [23].

### Cytokine measurements

Cytokine levels were measured at the Cytokine Core Laboratory (University of Maryland, Baltimore, MD) using ELISA and Luminex platforms.

### Chemotaxis assays

For the chemotaxis assay, we used the EZ-TAXIScan with 10% zymozan-activated serum as chemoattractant as previously described [25].

### Data analysis and statistics

Between group comparisons were performed by one way ANOVA followed by Bonferroni’s honestly significant difference post-hoc tests. Student t-test was performed when only two groups were compared. *P* < 0.05 was considered statistically significant. All data are presented as average with effort bars indicating the standard error of the mean.

## Results

### Irf6 is expressed in innate immune cells

**Fig 1:**
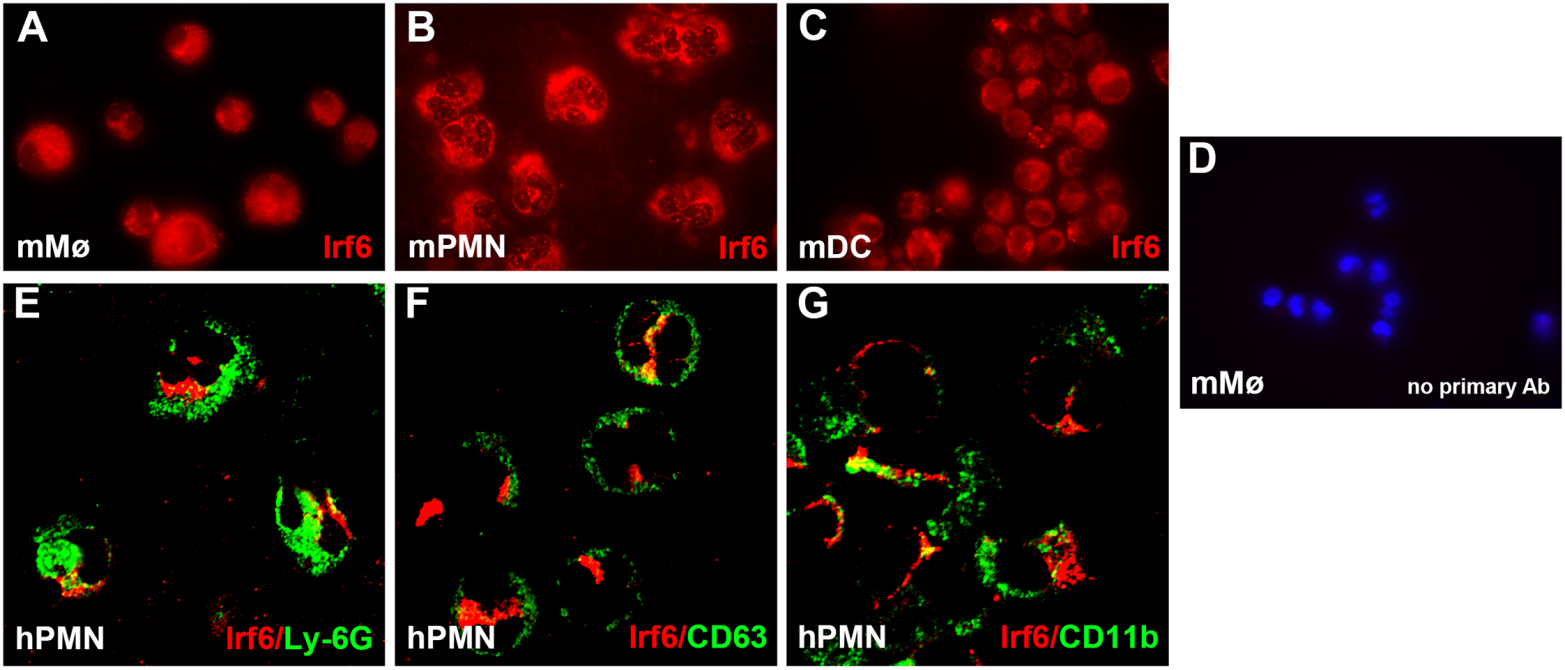
Irf6 is expressed in innate immune cells. (A-D) Murine bone marrow-derived macrophages (A, D, mMΦ), neutrophils (B, PMN) and dendritic cells (C, DC) were immunostained for Irf6 (red) or nuclear DAPI (blue). (E-G) Human blood-derived leucocytes (hPMN) were immunostained for Irf6 (red) and ly-6G (green, E), CD63 (green, F), and CD11b (green, G).

**Fig 2:**
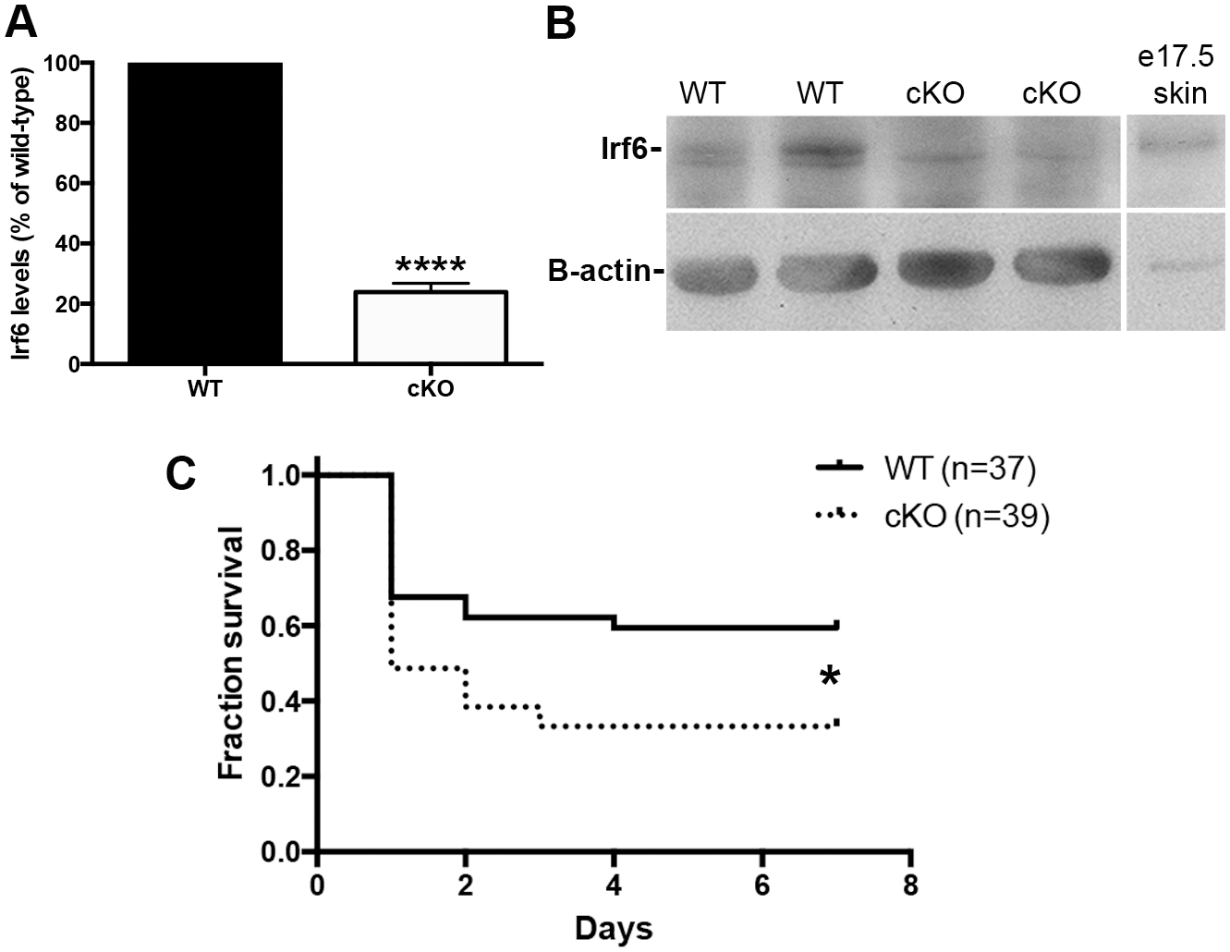
Irf6 in innate immune cells is required for resistance to LPS-induced endotoxic shock. (A) qPCR for *Irf6* in bone marrow-derived macrophages from wild-type and cKO animals. Data are expressed as percentage of wild-type (n = 3; unpaired t-test, **** *P* < 0.0001). (B) Western blot analysis of protein extracts from WT (n = 2) and cKO macrophages (n = 2), and embryonic day (e) 17.5 skin (C) Survival of wild-type and cKO was assessed over a 7-day period following LPS-induced endotoxic shock (n = 37–39, Gehan-Breslow-Wilcoxon test, * *P* < 0.05).

In a previous evaluation of the expression of Irf6 in murine cutaneous wounds, we detected Irf6 in keratinocytes at all stages of healing, and noticed the presence of Irf6-positive cells in the granulation tissue around 4 days post wounding. We speculated these cells could be innate immune cells based on histological analysis [20]. To confirm our hypothesis, we investigated the expression of Irf6 in isolated innate immune cells in the mouse. We detected Irf6 protein in macrophages, neutrophils and dendritic cells obtained from murine bone marrow (Figs 1A–1C and 2B). We also detected IRF6 protein in human neutrophils obtained from circulating blood (Fig 1E–1G). We did not examine the expression of IRF6 in human macrophages, but in all the cellular populations available to us, Irf6 was cytoplasmic, and did not colocalize with Ly-6G, CD63 or CD11b, all markers of different types of neutrophilic granules. It is worth noting that Ly-6G does not exist in human, therefore the signal that is detected in Fig 1E is likely the related molecule CD177, although it was not formally tested. These data suggest that Irf6 is present in innate immune cells, but is likely not secreted from cytoplasmic granules.

### LPS-induced endotoxic shock is dependent on Irf6

In order to define the function of Irf6 during the innate immune response *in vivo*, we crossed *Irf6*^*fl/fl*^ or *Irf6*^*fl/nl*^ mice with the *LysM*^*Cre*^ mice [27]. The resultant *LysM*^*Cre*^;*Irf6*^*fl/fl*^ or *LysM*^*Cre*^;*Irf6*^*fl/nl*^ (later referred to as Irf6 conditional knockout or cKO) mice had an expected Mendelian ratio at birth, and did not exhibit an apparent post-natal phenotype compared to wild-type animals (data not shown). We performed quantitative PCR for Irf6 in bone marrow-derived macrophages from wild-type and cKO animals. Our results show an 80% reduction in endogenous Ievels of *Irf6* mRNA in macrophages from cKO compared to cells from wild-type animals, (Fig 2A). Western blot data also support the presence of Irf6 in wild type macrophages and decreased levels in cKO cells (Fig 2B). These data confirm the efficient allelic recombination leading to a substantial reduction in Irf6 in bone marrow-derived macrophages.

Taking advantage of our unique murine models, we asked whether Irf6 was required for proper resistance to *in vivo* LPS-induced endotoxic shock. We injected 15 mg/kg body weight of LPS intraperitoneally in wild-type and cKO animals and assessed their survival daily over a 7 day period. The vast majority of wild-type animals survived following the LPS injection, with 70% of the animals being alive after a week. However, only 35% of cKO survived following LPS injection. Most of the death occurred within the first 48 h, indicating a quick physiological response most likely implying the innate immune system. Together, these data demonstrate that the presence of Irf6 in innate immune cells is required for proper resistance to LPS-induced endotoxic shock.

### Cytokine levels and leucocytic peritoneal migration were unchanged following LPS injection *in vivo*

**Fig 3:**
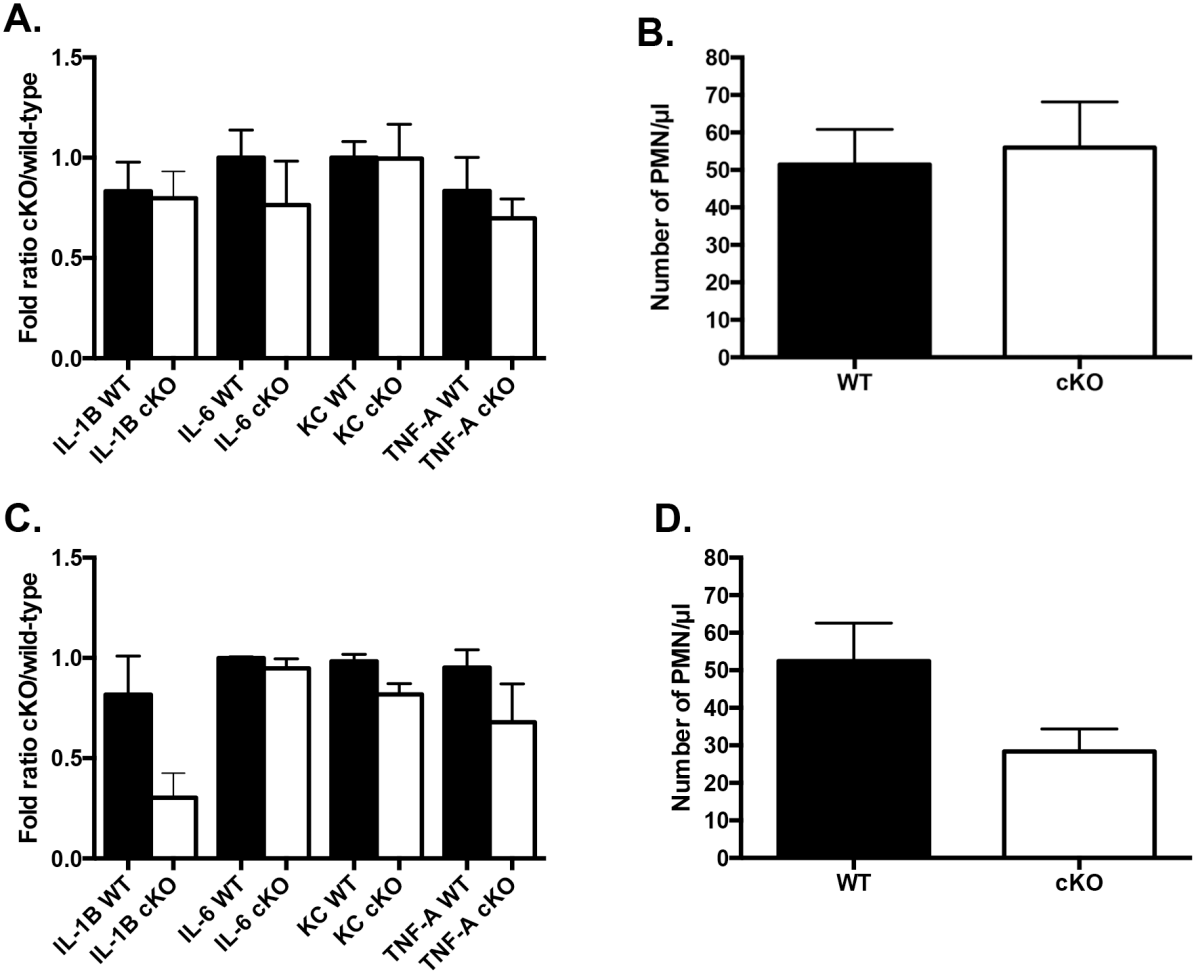
*In vivo* cytokine levels and peritoneal migration of neutrophils are not significantly different between wild-type and cKO. Low (LPS 15 mg/kg, A, B) and high (LPS 400 mg/kg, C, D) levels of LPS were injected intraperitoneally in wild-type (WT) and cKO animals. (A, C) Serum cytokine levels were measured 8 h following injection and reported as fold ratio cKO over wild-type. (B, D) Number of neutrophils (PMN) were counted following intraperitoneal lavage. Data are means ± SEM, n = 6–13 animals per group.

We further investigated the molecular mechanisms underlying the Irf6-dependent endotoxic shock response by measuring serum cytokine levels and leucocytic peritoneal migration. We collected serum from wild-type and cKO animals following intraperitoneal LPS injection. Regardless of the amount of LPS injected (15 and 400 mg/kg body weight), levels of interleukin (IL)-1B, IL-6, KC and TNF-α were similar in both groups (Fig 3A and 3C). Furthermore, the number of neutrophils in the intraperitoneal cavity 8 h following LPS injection was not significantly different between wild-type and cKO animals (Fig 3B and 3D). These data suggest that peritoneal migration of neutrophils is not Irf6-dependent.

### Neutrophil chemotaxis *in vitro* is Irf6-dependent

**Fig 4:**
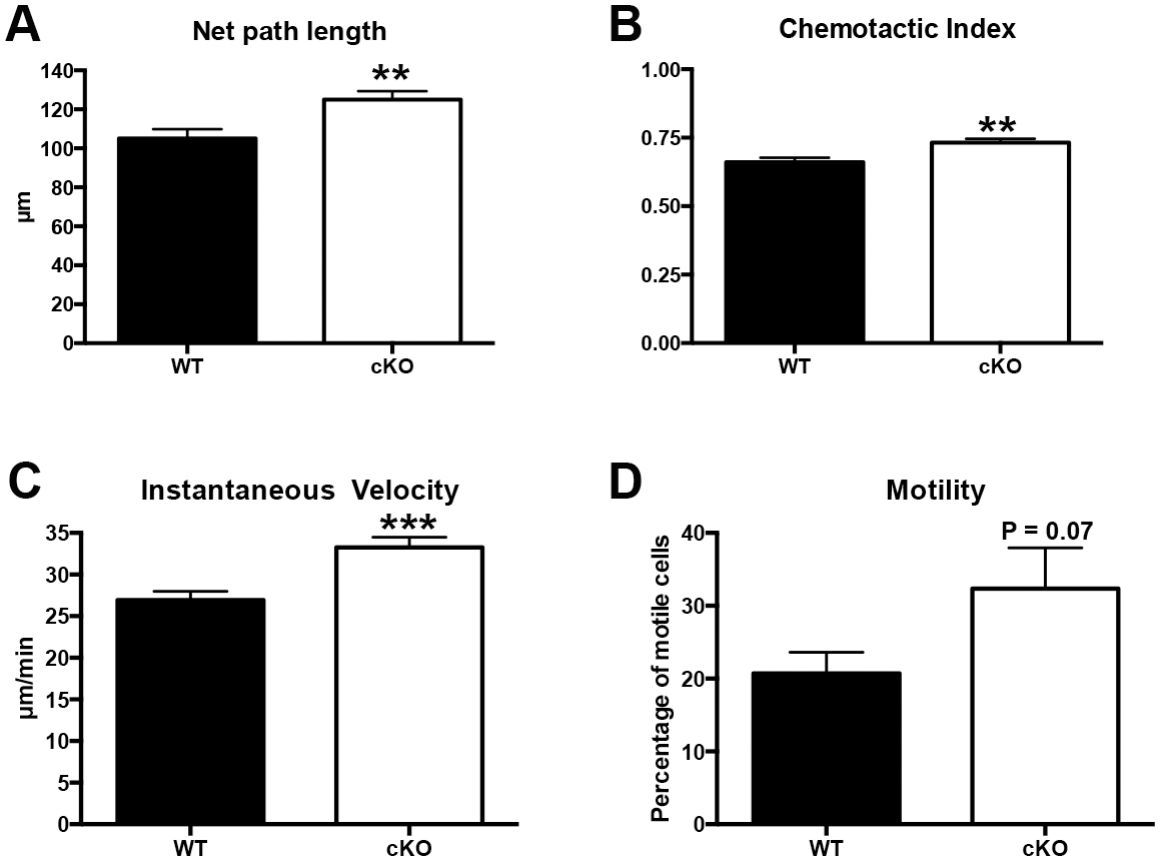
Irf6 promotes *in vitro* migration of neutrophils as determined by the EZ-TAXIScan assay. Murine bone marrow-derived neutrophils were stimulated with an optimal ZAS spatial gradient generated by a 10% ZAS loading concentration using the EZ-TAXIScan chamber. (A) Net path length (μm), (B) Chemotactic index (arbitrary units), (C) Instantaneous velocity (μm/min), and (D) Percentage of motile cells in the tested cell population. Data are means of 7 experiments (150–250 cells total per group) ± SEM. ** *P* < 0.001, *** *P* < 0.0005 following t-test.

We previously demonstrated the requirement for Irf6 in keratinocyte migration *in vitro* using a scratch wound assay [20]. Therefore, we asked whether Irf6 would play a similar role in neutrophils by dissecting out the multiple facets of neutrophil chemotaxis *in vitro*. Using an EZ-TAXIScan *in vitro* system, we followed the chemotaxis of wild-type and cKO murine bone marrow-derived neutrophils using 10% zymozan-activated serum (ZAS) as chemoattractant (Fig 4). This model was chosen as it showed the best neutrophil chemotaxis response [25]. Our results demonstrated a significant increase in net path length, chemotactic index and instant velocity in neutrophils from cKO animals compared to wild-type (Fig 4A–4C). The motility was also increased, although the data was not significant (*P* = 0.07). These data suggest that in the absence of Irf6, neutrophils are faster and respond more quickly to ZAS, suggesting that Irf6 negatively regulates chemoattraction of bone marrow-derived neutrophils *in vitro*.

### IRF6 negatively regulates cytokine production *in vitro*

**Fig 5:**
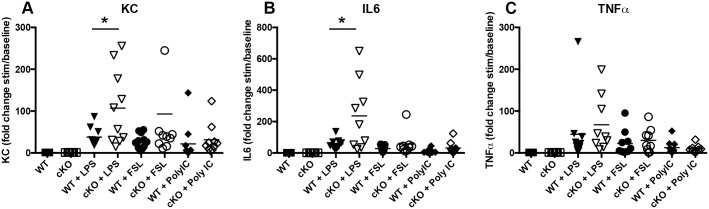
Irf6 inhibits cytokine production in bone marrow-derived macrophages *in vitro*. Cytokines were measured in conditioned medium from unstimulated (baseline) macrophages, or macrophages stimulated with LPS (50 ng/ml), FSL (1 μg/ml) or Poly IC (10 μg/ml) from wild-type (WT) and Irf6 cKO (cKO). Data are expressed as fold change between stimulated conditioned medium over baseline. N = 9–11 per group. Each symbol represents a biological replicate, horizontal bar represent the mean. * *P* < 0.05.

In addition to neutrophils, macrophages are another critical contributor to the innate immune response. In order to determine the Irf6-dependent regulation of cytokine production in macrophages, we cultured wild-type and cKO bone marrow-derived macrophages *in vitro*. Cells were stimulated with different TLR-specific agonists: LPS (TLR-4), FSL (TLR-2 and TLR-6) and polyIC (TLR-3). KC, IL6, and TNFα levels were measured in the culture supernatant 8 h after stimulation. As shown in Fig 5, the presence of LPS induced a significant increase in KC (Fig 5A) and IL6 (Fig 5B) production in the supernatant of cKO macrophages compared to supernatant from wild-type cells. A similar trend was observed with TNFα, but was not significant (Fig 5C). The presence of FSL-1 or PolyIC did not significantly alter the level of cytokines in conditioned medium from either group of macrophages, suggesting that this effect is LPS specific. As LPS specifically activates the TLR-4 pathway, together, these data indicate a TLR4-mediated Irf6-dependent repression of KC and IL6 in bone-marrow derived macrophages.

### NFkB activation is Irf6-dependent

**Fig 6:**
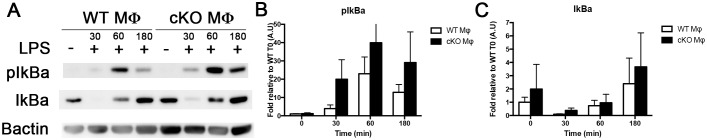
Irf6 inhibits NFkB activation in bone marrow-derived macrophages *in vitro*. (A) Western blot analysis (one representative of 3 experiments) for pIkBa, IkBa and Bactin of wild-type (WT) and Irf6 cKO (cKO) macrophages (MΦ) unstimulated or stimulated with LPS for 30, 60 and 180 min. (B, C) Quantitative analysis of three independent Western blot analysis as presented in (A) expressed as fold change from the unstimulated (T0) control cells.

Toll-like receptors (TLR) are critical to the detection and the innate immune response to pathogens. TLR signaling activates a cascade of downstream mediators that ultimately lead most commonly to the translocation of NFkB to the nucleus to activate gene expression [28]. In order to enter the nucleus, NFkB has to be released from its IkB-bound complex in the cytoplasm. This will occur upon phosphorylation of IkB, releasing NFkB and targeting p-IkB for proteosomal degradation. Therefore, we evaluated the level of IkBa and p-IkBa following LPS stimulation *in vitro* to ask whether the NFkB signaling following TLR signaling was Irf6-dependent. Following LPS stimulation, the level of p-IkBa increased after 30 min to peak at 60 min and return to above baseline level at 3 h in wild-type macrophages (Fig 6). In cKO macrophages, however, the level of p-IkBa stayed high following peak expression (Fig 6). Simultaneously, levels of IkBa decreased 30 min after LPS stimulation and returned to near baseline levels after 60 min in wild-type macrophages (Fig 6). A similar pattern was observed in cKO macrophages (Fig 6). Although these data were not statistically significant, there appears to be a trend for more robust phosphorylation of IkBa in the absence of Irf6. Phosphorylation of IkBa results in the dissociation of the complex formed with NFkB, allowing the translocation of NFkB to the nucleus and thus, the activation of its gene targets. One target of NFkB is IkBa, forming a feedback loop that may explain the higher levels of IkBa in the cKO in unstimulated cells and at 3h post-stimulation. These data reinforce a potential anti-inflammatory role for Irf6.

## Discussion

In the present study, we report the presence of Irf6 in neutrophils, macrophages and dendritic cells. We demonstrate that Irf6 is required for proper resistance to LPS-induced endotoxic shock. Furthermore, Irf6 negatively regulates TLR4-mediated cytokine production, inhibits NFkB activation in bone marrow-derived macrophages *in vitro* and acts negatively on neutrophil motility. Our findings thus reveal new insights into the molecular mechanisms through which Irf6 contributes to endotoxic shock, suggesting that Irf6 has anti-inflammatory properties. In addition, it highlights a function for Irf6 in innate immunity, a role so far reserved for the other IRF family members.

The conditional removal of Irf6 in innate immune cells did not affect the percentage of the respective hematopoietic cell populations including leucocytes, monocytes, neutrophils white blood cells and red blood cells (data not shown), excluding the possibility that the lack of resistance to LPS-induced endotoxic shock is due to a deficiency in one of the cell population. However, we cannot rule out that Irf6 expression levels affect the differentiation of monocytes into macrophages, rendering these cells less competent in resisting endotoxic shock.

There is increasing evidence that TLRs play an essential role in mediating the signaling cascade leading to endotoxic shock [29]. Amongst the TLRs, TLR4 is the principal receptor for LPS leading to the activation of NFkB and the synthesis of cytokine production [30]. Our study demonstrates that Irf6 is a specific negative regulator of TLR4 in macrophages and controls the production of KC and IL6. This is in contrast to the recent described function of Irf6 in the epithelial cell line OKF6, in which it differentially regulates TLR2-dependent KC [31]. These findings suggest that Irf6 controls several TLRs in a cell-type-specific manner.

Although we observed a two-fold increase in susceptibility to endotoxic shock in our Irf6 cKO model compared with wild type animals, we did not detect differences in cytokine levels in blood serum. Several reasons could account for this observation. First, our cytokine measurement was performed 8 h following LPS induction, at which point all of our animals were still alive. Second, the amount of LPS (15 mg/kg body weight) was within doses that lead to a saturated system and maximum cytokine response. It may be, that the more important question is whether the response of the knockout decreases as fast as the one in the wild type. In other words, would the lingering inflammatory effect damage the knockout leading to incomplete recovery and death? To test this question, one must harvest samples at 12 h and 18 h post LPS injection, which represents technical challenges as the mice are in endotoxic shock and their blood would be hard to harvest.

Innate immune cells are characterized by their ability to respond quickly to non-self. Although the number of neutrophils migrating into the peritoneal cavity 8 h after LPS injection was similar between Irf6 cKO and wild-type animals, we detected differences using the *in vitro* EZ-TAXIScan. Similar discrepancies between *in vivo* and *in vitro* neutrophils migration were observed previously [32]. They may reflect the point that peritoneal elicitation of neutrophils is distinct from neutrophil chemotaxis. In fact, migration of cells in the peritoneal cavity would probably be better described as transendothelial migration through the peritoneal membrane, which includes excavation of cells from the vasculature. Furthermore, the *in vivo* migration of neutrophils is not very sensitive for detecting differences in individual cell migration, since some cells could come rushing in early and others could be late. If the total number of migrating cells is similar, a migration defect could be easily missed. Such differences, however, are detectable with the EZ-TAXIScan system.

Indeed, we found cKO neutrophils overall faster and more motile compared with wild-type cells. This is in contrast with our previously published results with murine keratinocytes. Using an *in vitro* scratch-wound model, we found that Irf6-deficient keratinocytes were impaired at closing the gap [20]. Our studies with keratinocytes demonstrated that Irf6 was required for regulating cell-matrix adhesion; in the absence of Irf6, cells were more spread, more “sticky” and exhibited increased stress fibers. Consequently, Irf6-deficient keratinocytes were slower at migrating to close the scratch. The EZ-TAXIScan, on the other hand, involves very little adhesion of neutrophils to a substrate. Therefore, the increased velocity and motility of Irf6 cKO neutrophils could be due to their activated state making them more sensitive to chemokines.

Our data suggest an inhibitory role for Irf6 in regulating TLR-dependent pathways. One other IRF (IRF4) has been described as a negative regulator of TLR signaling [33]. Most of them seem to target the MyD88 signaling cascade [5]. MyD88, along with IRAK1, activates Irf6 in epithelial cell lines [22]. We have not investigated, in this level of detail, the signaling cascade of neutrophils and macrophages deficient for Irf6. However, we evaluated the NFkB transcriptional machinery via the phosphorylation state of IkBa and found sustained p-IkBa following LPS induction. Our results are consistent with a role for Irf6 in negatively controlling the proinflammatory cytokine production as previously described [28]. Irf6 could be a regulatory molecule necessary to shut down the immune response by controlling the amount of cytokine that is produced, but also by sending a signal through the more global NFkB pathway. It is interesting to note that mice deficient for *Chuk* (also called IKKa), another component of the NFkB pathway, share a similar phenotype to the *Irf6* deficient animals. Moreover, genetic mutations in *IRF6* or *CHUK* have been identified in individuals clinically diagnosed with Bartsocas-Papas syndrome, a form of popliteal pterygium syndrome [34], supporting the global notion that Irf6 and components of the NFkB pathway are part of the same gene regulatory network.

Collectively, our data support the overall concept that in the absence of Irf6, neutrophils and macrophages are attracted to sites of injury and activated to secrete cytokines. This could be detrimental to life when generalized to the entire body, as observed in our septic shock model. However, at the tissue level, this could be beneficial to fight local infection. In an evolutionary perspective, one could envision that carrying a genetic variant in *IRF6* promoting local secretion of cytokines would confer a selection advantage by exhibiting anti-inflammatory properties and protecting against infectious agents. It was previously reported that one third of the population carries a genetic variant in an *IRF6* enhancer associated with increased risk for cleft lip and palate [35]. It would be interesting to further investigate a potential association between this variant and severity of illness in septic patients or post-surgical wound healing outcome to identify patients at high risk for development of disease.
