# Correction: Interferon Regulatory Factor 6 Has a Protective Role in the Host Response to Endotoxic Shock

**DOI:** 10.1371/journal.pone.0171459

**Published:** 2017-01-30

**Authors:** Sophie Joly, Lindsey Rhea, Paige Volk, Jessica G. Moreland, Martine Dunnwald

In the Funding section, the second grant number from the funder National Institutes of Health is listed incorrectly. The correct grant number is: AR067739.
